# Hypoxia inducible factor 1 subunit alpha mediates autophagy disorder of oral lichen planus by regulating lysosomal pathway

**DOI:** 10.3389/fimmu.2026.1789261

**Published:** 2026-04-14

**Authors:** Xie Li, Chao Han, Yuemei Yao, Yuanxin Shi, Bin Chen, Yi Xiao, Guohui Bai

**Affiliations:** 1 Key Laboratory of Oral Diseases Research, School of Stomatology, Zunyi Medical University, Zunyi, Guizhou, China; 2 Institute of Life Sciences, Zunyi Medical University, Zunyi, Guizhou, China; 3 The Key Lab of Guizhou Provincial Department of Education for Medical Prevention and Treatment of Tumor, Zunyi Medical University, Zunyi, Guizhou, China

**Keywords:** autophagy, HIF-1α, lysosome, oral lichen planus, *Porphyromonas gingivalis* lipopolysaccharide

## Abstract

Oral lichen planus (OLP), known as a common and chronic mucosal inflammatory disease. Due to its potential for cancerous transformation, this disease has long been a subject of significant concern. Its pathogenesis is that an unknown antigen activates oral keratinocytes and antigen-presenting cells (APCs) in the epithelium, leading to a T cell-mediated immune response. This study investigates the regulatory role of HIF-1α transcription factor and the potential role of autophagy in a simulated OLP inflammatory cell model. In this study, by comparing the control group with the model group, we observed a notable up-regulation of *HIF1A* mRNA expression. Additionally, both the autophagy marker protein LC3-II and its substrate p62 exhibited abnormal accumulation, suggesting that autophagy is impaired at the lysosomal degradation stage. Furthermore, mechanistic studies have shown that HIF-1α leads to dysfunction of the autophagy-lysosomal pathway by inhibiting the expression of lysosomal-related genes, while knockdown of HIF-1α alleviates the disruption of autophagic and promotes the improvement of cell activity. In experiments with *C. elegans*, stimulation with *Porphyromonas gingivalis* lipopolysaccharide (*P. gingivalis* LPS) shortens worms’ lifespan, but knocking down the *hif-1* gene reverses this effect. Similarly, deletion of the key autophagy genes *bec-1* and *lgg-1* reduces worm survival rates. Results indicate that HIF-1α plays a vital role in regulating autophagy-lysosomal function and cellular homeostasis. In summary, the excessively high expression of HIF-1α aggravates cellular and systemic damage by impairing autophagy-lysosomal function in OLP. Conversely, the targeted inhibition of HIF-1α restores autophagy function, suggesting a potential therapeutic strategy.

## Introduction

Oral lichen planus (OLP) is a skin-mucosa chronic inflammatory disease (1), which is one of the most common oral mucosal diseases. The prevalence in the general population is about 1-2%, but the incidence is highest in women aged 30-60 years old (2). It is defined as a potential malignant disease by the World Health Organization (WHO), the malignant rate is 0% to 3.5% (3), and most of them are transformed into oral squamous cell carcinoma. As so far, there remains no cure for OLP, and the primary treatment approach is symptomatic control. Its pathological features primarily include lymphocytic infiltration of the lamina propria, degeneration of keratinocytes, and destruction of the basement membrane (4). The specific pathogenesis remains unclear, but evidence suggests that innate immune cells and oral keratinocytes mount a chronic, dysregulated immune response to antigens mediated by OLP, which eventually leads to oral keratinocyte death, mucosal basement membrane destruction and long-term chronicity of the disease (5).

Autophagy is a conserved cellular lysosomal degradation process that is essential for maintaining cellular homeostasis and normal development, characterized by autophagosome formation, autophagosome-lysosome fusion, and autophagolysosomal degradation (6). Under numerous pathophysiological conditions, defects in the endolysosomal autophagy pathway caused by altered lysosomes have been extensively investigated in fields such as autoimmune diseases, metabolic disorders, and renal diseases (7). In OLP, it has been reported that the expression of cathepsin K, a member of the lysosomal protease family, is significantly up-regulated and is closely related to inflammatory activation (8). This may be related to changes in lysosomal function, but this potential interaction remains to be further investigated. On the other hand, the dysregulation of autophagy-related genes is believed to increase susceptibility to a variety of diseases, including inflammation, autoimmune diseases and cancer (9, 10). Previous studies have found that the expression of *unc-51* like autophagic activating kinase (ULK1) and microtubule-associated protein 1 light chain 3 (LC3) is increased in T cells of OLP (11), this suggests that dysregulated T cell autophagy may contribute to the immune response in OLP and may also correlate with clinical patterns (12). However, it remains unclear whether other mechanisms are involved in regulating autophagy under the immune-inflammatory conditions of OLP.

Hypoxia-inducible factor 1 alpha (HIF-1α) is a subunit of the HIF-1 transcription factor (13). Under normoxic conditions, the HIF-1α subunit undergoes rapid degradation by the ubiquitin-proteasome system. In fact, HIF-1α protein expression is generally low in normoxic cells within normal human tissues, but is highly expressed in hypoxic cells (14). Therefore, activation of HIF-1α is an important method for hypoxic cells to adapt to hypoxic tension in harsh microenvironment. Numerous studies indicate that HIF-1α, as a key transcription factor under hypoxic conditions, regulates angiogenesis, glucose metabolism, apoptosis, and autophagy, participating in the control of multiple signaling pathways (15, 16). And in different cell types, it participates in regulating autophagy at varying levels, effectively alleviating abnormal autophagy and apoptosis in cells (17–19). Similarly, studies have found that the expression of HIF-1α may play an important role in the chronicity of oral mucosal lesions in OLP (20, 21).

Our investigation revealed that dysregulated lysosomal function and autophagy in oral lichen planus (OLP). Then, the prediction analysis of transcription factors found that HIF-1α may influence autophagy levels by regulating lysosomal function, thereby modulating cellular autophagy homeostasis. After knocking down HIF-1α, autophagy returned to normal, and the mRNA levels of lysosomal pathway genes gradually decreased to normal levels. These results implicate HIF-1α in OLP progression through its modulation of the autophagy pathway.

## Results

### The activity of HOK cells decreased in oral lichen planus, accompanied by an increase in inflammatory factors

**Figure 1:**
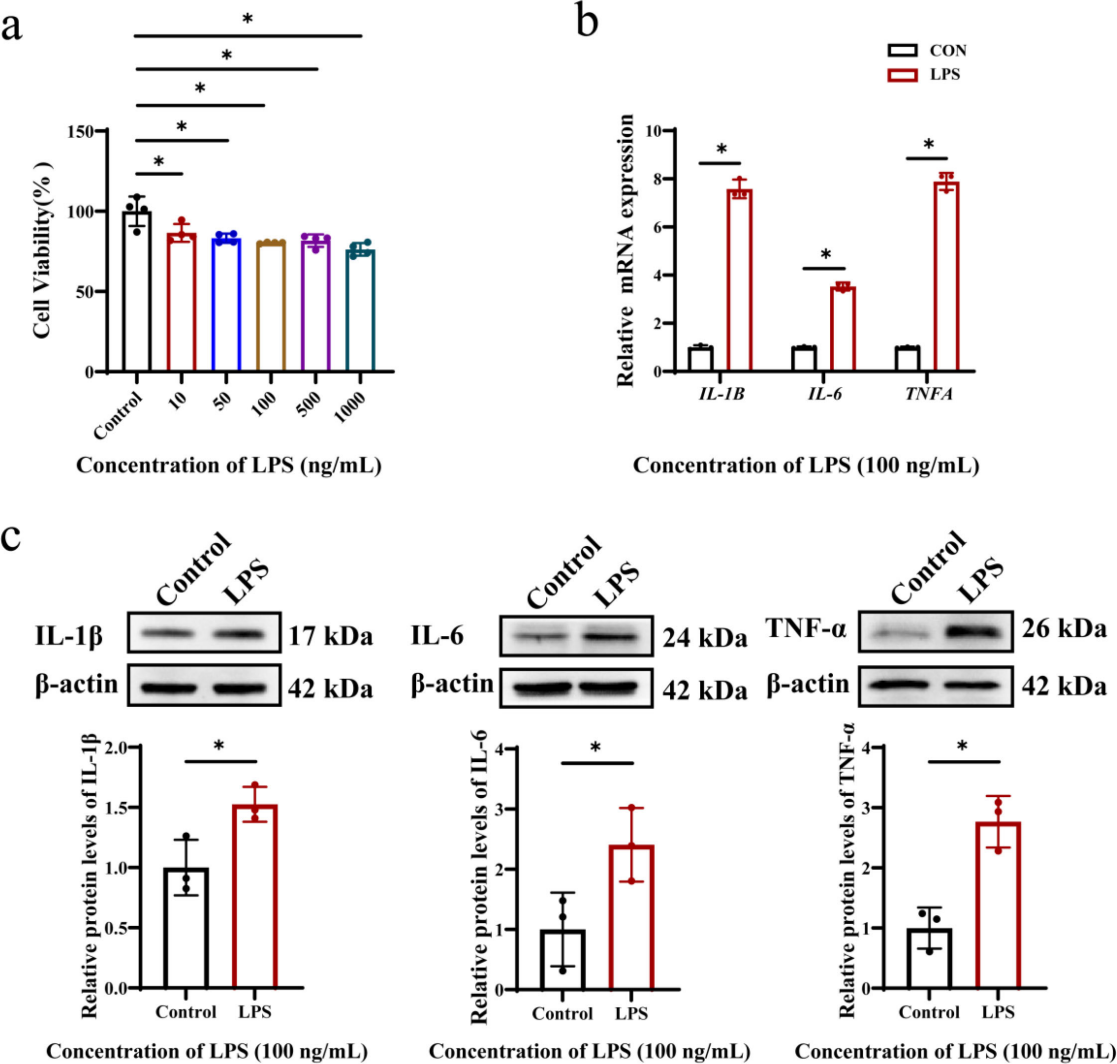
Establishment of cell inflammation model of oral lichen planus. **(a)** Compared with the control group, the activity of cells treated with *P. gingivalis* LPS was suppressed. Data are expressed as mean ± SD. *P* values were calculated using one-way analysis of variance (ANOVA). **(b)** The mRNA levels of inflammatory factors in the *P. gingivalis* LPS group increased. **(c)** And the expression of inflammatory factor proteins in the *P. gingivalis* LPS group increased. Data were expressed as mean ± SD. The *P* value was calculated using the independent sample t test for the control group. *P < 0.05. All assays were conducted for three biological times, n = 3.

To construct the cell inflammation model of oral lichen planus (22), we used different concentrations (10 ng/mL, 50 ng/mL, 100 ng/mL, 500 ng/mL, 1000 ng/mL) of *Porphyromonas gingivalis* lipopolysaccharide (*P. gingivalis* LPS) to stimulate human oral keratinocytes (HOK). Cell Counting Kit-8 (CCK-8) assay results showed that cell viability decreased in a concentration-dependent manner and tended to be stable at 100 ng/mL (**Figure 1A**). Therefore, this concentration is used for subsequent experiments. The results of RT-qPCR and Western Blotting showed that compared with the control group, *P. gingivalis* LPS stimulation significantly up-regulated the mRNA levels and the protein levels of IL-1β, IL-6 and TNF-α in HOK cells (**Figures 1B, C**). These results confirmed that *P. gingivalis* LPS inhibited the activity of HOK cells and induced inflammation in HOK cells.

### Abnormalities in lysosomal are present in oral lichen planus

**Figure 2:**
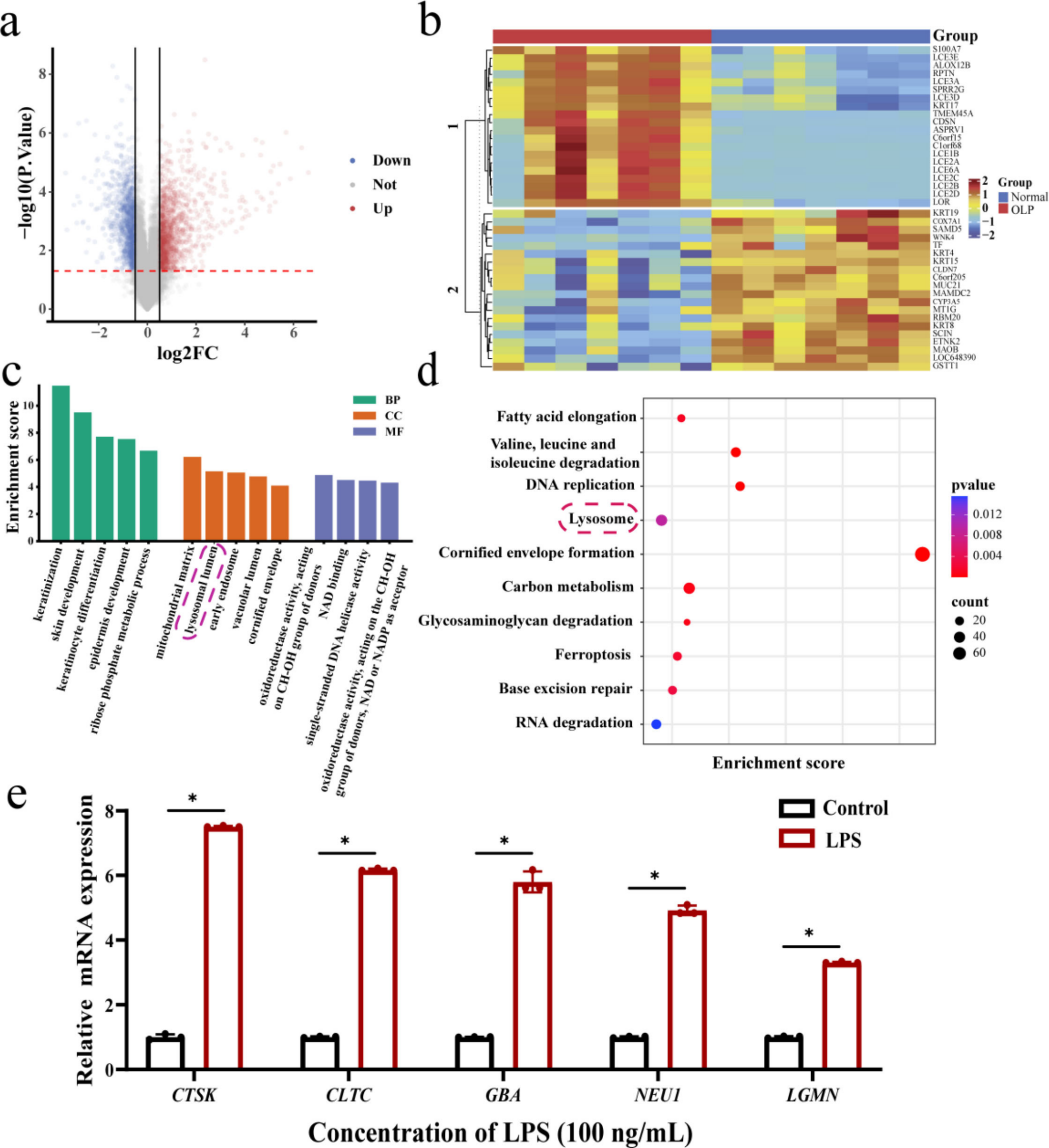
Abnormal expression of lysosomal pathway genes. **(a)** Volcano diagram, abscissa represents log2 difference change multiples, ordinate is -log10(*P*-value). **(b)** Heat map, up and down the differential gene expression of each top 20. **(c)** GO enrichment classification map. **(d)** KEGG enrichment classification map. **(e)** The mRNA levels of lysosomal pathway genes in HOKs with *P. gingivalis* LPS-treated. Data were expressed as mean ± SD. The *P* value was calculated using the independent sample t test for the control group. *P < 0.05. All assays were conducted for three biological times, n = 3.

According to the screening conditions of the data set: sufficient sample size, reasonable group setting to meet statistical requirements, and data based on high-throughput platforms (such as Affymetrix or Illumina chip), we selected the data set GSE52130. Additionally, this dataset has been used multiple times and is suitable as a benchmark dataset. Subsequently, the microarray expression dataset was downloaded from the GEO database and used the limma package in R to compare the differences in gene expression levels between OLP samples and Control samples within the GEO dataset GSE52130. The screening conditions were: |log2FC| > 0.5, *P* < 0.05. GSE52130 screened 2427 differentially expressed genes (DEGs), of which 1182 were up-regulated and 1245 were down-regulated. The DEGs were visualized using volcano maps and heat maps (**Figures 2A, B**). Subsequently, Gene Ontology (GO) and Kyoto Encyclopedia of Genes and Genomes (KEGG) analysis were performed to reveal the potential biological functions and enrich pathways of the DEGs. GO enrichment analysis was performed with a threshold of adjusted *P* (FDR) < 0.05, identifying 66 significantly enriched entries. Among these, 53 entries were enriched in biological processes (BP), 9 in cellular components (CC), and 4 in molecular functions (MF). BP of DEGs is mainly enriched in cell cycle, cell proliferation and cell death regulation, epithelial cell differentiation regulation and stress response to maintain cell homeostasis. Meanwhile, CC primarily accumulates in the mitochondrial matrix, lysosomal lumen, and early endosomes/early endosomal membranes. The lysosomal lumen is responsible for intracellular clearance and recycling, playing a crucial role in maintaining cellular homeostasis, immune defense, and programmed cell death. In MF, DEGs are mainly enriched in energy metabolism and genetic information reading (**Figure 2C**). The screening condition of KEGG enrichment analysis was *P* < 0.05, and a total of 58 significant entries were enriched. Similarly, we enriched the lysosomal pathway (**Figure 2D**). And RT-qPCR revealed that genes along this pathway exhibited an overall up-regulation trend in *P. gingivalis* LPS treated HOK cells compared to untreated HOK cells (**Figure 2E**). These findings suggest that lysosomal normality is required for OLP.

### HIF-1α may participate in regulating the expression of genes associated with the lysosomal pathway in oral lichen planus

**Figure 3:**
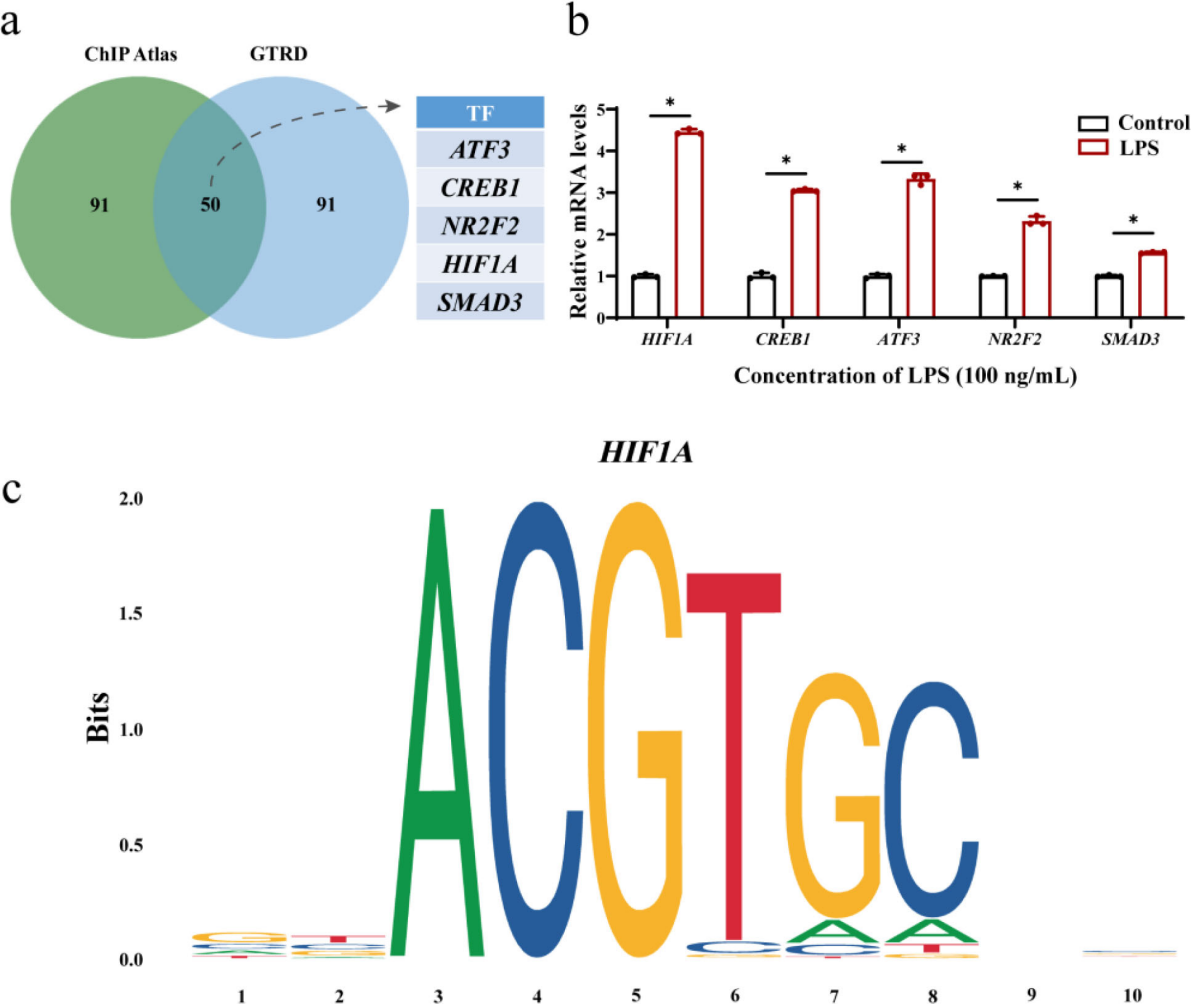
Transcription factor prediction and verification. **(a)** A total of 50 overlapping transcription factors were obtained. **(b)** The expression of *ATF3, CREB1, NR2F2, HIF1A*, and *SMAD3* mRNA levels in the *P. gingivalis* LPS treatment group was up-regulated. Data are presented as mean ± SD. The *P* value was calculated using the independent sample t test for the control group. **(c)** *HIF1A* sequence logo. *P < 0.05. All assays were conducted for three biological times, n = 3.

In OLP, is there an upstream gene that regulates this series of lysosomal genes? Therefore, we conducted a transcription factor analysis and submitted the genes on the lysosomal pathway to the online database CHIP Atlas and GTRD. The analysis results obtained a total of 50 intersecting transcription factors (**Supplementary Table 1**). The five factors of *ATF3*, *CREB1*, *NR2F2*, *HIF1A* and *SMAD3* caught our attention (**Figure 3A**). RT-qPCR results indicate that the mRNA levels in *P. gingivalis* LPS treated HOK cells showed degrees of elevation compared to the control group, with the most pronounced difference observed in HIF-1α expression (**Figure 3B**). Then, we visualize its sequence logo of transcription factor HIF-1α (**Figure 3C**). These results suggest that HIF-1α may participate in regulating the expression of genes associated with the lysosomal pathway in oral lichen planus.

### Knockdown of HIF-1α down-regulates genes highly expressed in the lysosomal pathway and restores cellular activity

**Figure 4:**
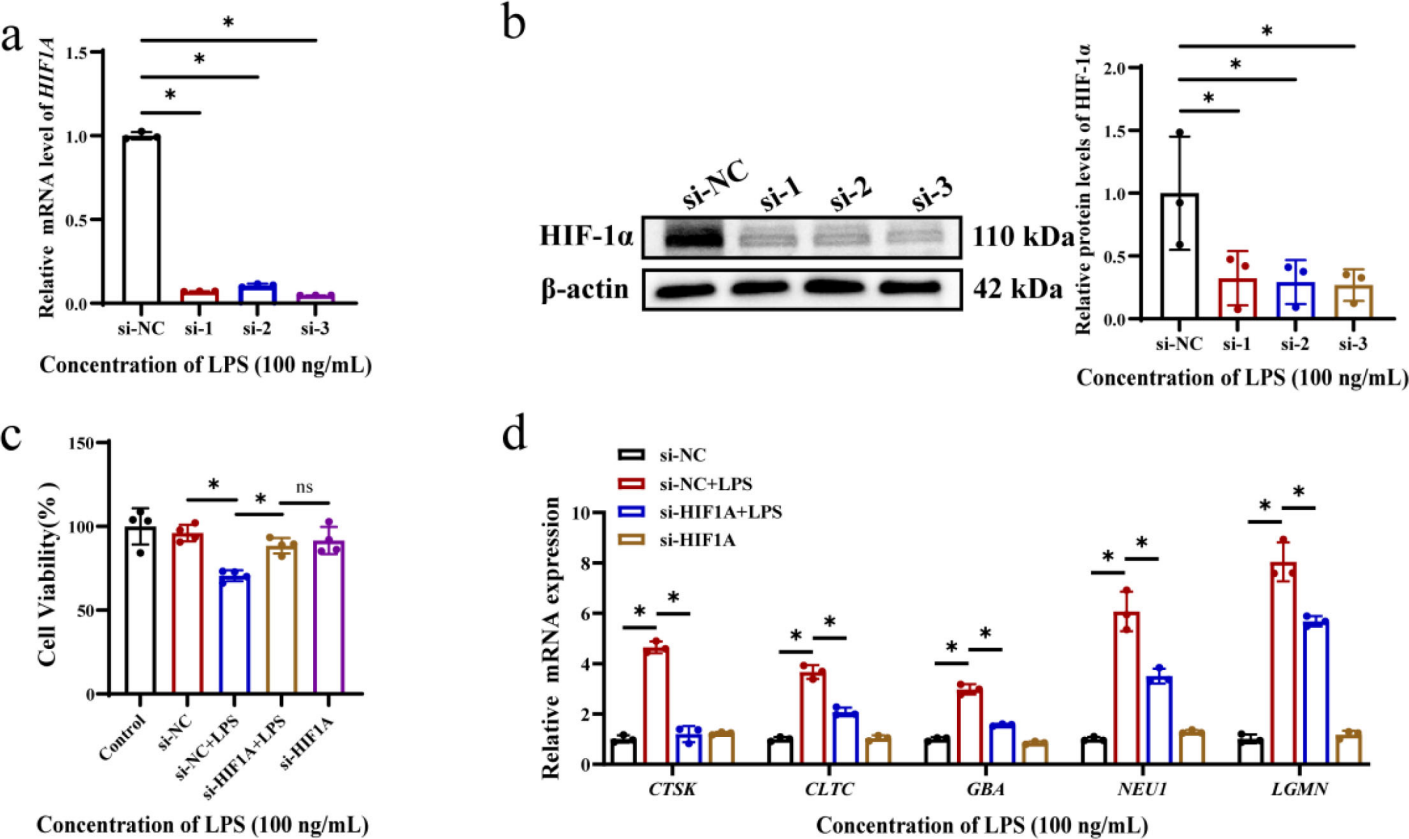
Knockdown of HIF-1α significantly down-regulated most of the abnormally highly expressed genes in the lysosomal pathway. **(a)** The mRNA levels of *HIF1A* at 24 h after knockdown. **(b)** The protein levels of HIF-1α after 48 h of knockdown. **(c)** The activity of HIF-1α knockdown HOKs was not inhibited after *P. gingivalis* LPS administration. **(d)** After *P. gingivalis* LPS administration, the mRNA levels in the lysosomal pathway of HIF-1α knockdown HOKs were gradually restored compared with the transfected control group. Data are presented as mean ± SD. One-way analysis of variance (ANOVA) was used to calculate *P* values. *P < 0.05. ns (no significance). All assays were conducted for three biological times, n = 3.

To test the knockdown efficiency of HIF-1α, we detected the transcription levels and protein levels respectively. The results showed that si-1, si-2 and si-3 could significantly reduce the expression of HIF-1α (**Figures 4A, B**), so the optimal si-3 was selected for subsequent experiments. Then, the HOK cells of knockdown HIF-1α were treated with *P. gingivalis* LPS, and it was found that the cell activity of the si-NC + LPS group was significantly lower than that of the si-HIF1A + LPS group (**Figure 4C**), suggesting that the high expression of HIF-1α may be an important factor leading to the decrease of HOK cells activity in OLP. At the same time, the mRNA levels of highly expressed lysosomal pathway-related genes gradually returned to normal after HIF-1α knockdown (**Figure 4D**). These results indicate that the abnormal overexpression of HIF-1α in OLP may be closely related to the up-regulation of lysosomal genes, and interference with HIF-1α expression can partially restore cell activity.

### Up-regulation of lysosomal genes can induce abnormal autophagy, while HIF-1α knockdown can reverse this effect

**Figure 5:**
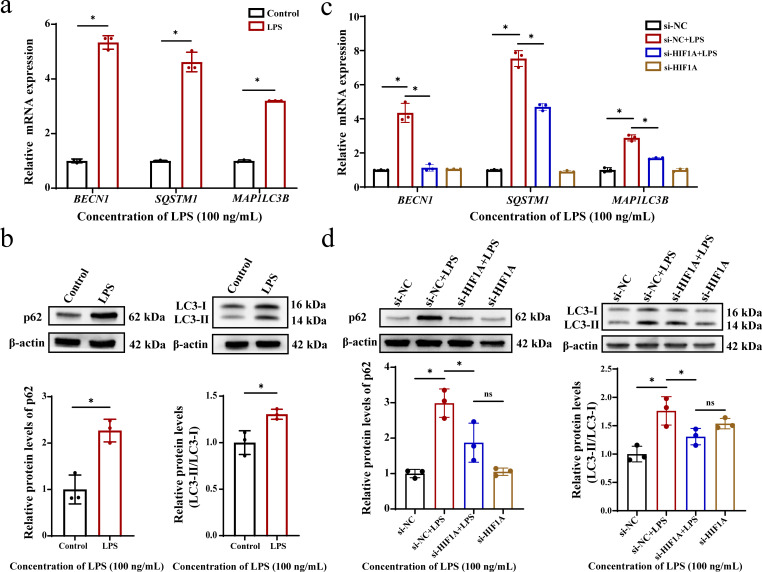
Lysosomal genes up-regulation caused autophagic defects, which were reversed by HIF-1α knockdown. **(a)** The mRNA levels of *BECN1*, *SQSTM1*, and *MAP1LC3B* in HOK cell with *P. gingivalis* LPS-treated. **(b)** The protein levels of p62 and LC3 in HOKs with *P. gingivalis* LPS-treated. Data are presented as mean ± SD. The *P* value was calculated using the independent sample t test for the control group. **(c)** After *P. gingivalis* LPS administration, the mRNA levels of *BECN1*, *SQSTM1*, and *MAP1LC3B* in the HIF-1α knockdown group. **(d)** After *P. gingivalis* LPS administration, the protein levels of p62 and LC3 in the HIF-1α knockdown group. Data are presented as mean ± SD. One-way analysis of variance (ANOVA) was used to calculate *P* values. *P < 0.05. ns (no significance). All assays were conducted for three biological times, n = 3.

Lysosomes, as single-layer membrane-bound organelles containing acidic hydrolases, play a central role in degrading intracellular substances and maintaining cell homeostasis. It not only participates in multiple autophagy processes, such as microautophagy, macroautophagy, and chaperone-mediated autophagy, but also engages in various cell death pathways including lysosomal cell death, apoptosis, and autophagic cell death. Due to the wide range of lysosomal functions, whether its dysfunction affected the autophagy process in oral lichen planus remained unknown, we quantified the expression of the autophagy-related genes *BECN1*, *SQSTM1*, and *MAP1LC3B*. The results showed that compared with the control group, these genes were all up-regulated to various degrees in *P. gingivalis* LPS treated HOK cells (**Figure 5A**). At the same time, western blot analysis revealed that both the protein levels of p62 and LC3-II significantly increased under *P. gingivalis* LPS treatment conditions (**Figure 5B**). Collectively, these findings indicate the presence of impaired a autophagy in the OLP. Then, we investigated the role of HIF-1α in this process. After knocking it down, the mRNA levels of *BECN1*, *SQSTM1*, and *MAP1LC3B* were restored to nearly normal levels (**Figure 5C**). Consistent with this, the protein accumulation of p62 and LC3-II was significantly reduced (**Figure 5D**). The results show that the up-regulation of lysosomal genes led to abnormal autophagy in OLP, and knockdown of HIF-1α could effectively reverse this process, indicating that HIF-1α may be a key regulator mediating the pathological development of OLP.

### *P. gingivalis* LPS shortens the lifespan of *C. elegans*

**Figure 6:**
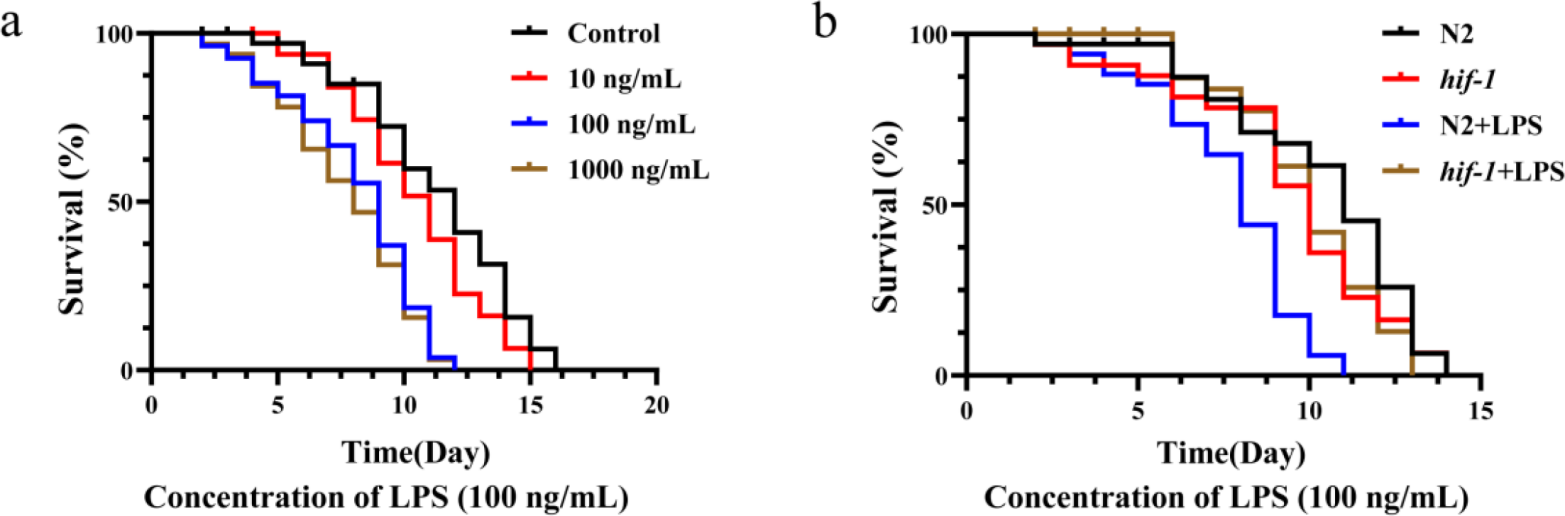
P*. gingivalis* LPS could shorten the lifespan of *C. elegans*, while the lifespan of *hif-1* mutant was not significantly reduced. **(a)** Survival of N2 hermaphrodite worms exposed to *P. gingivalis* LPS (100 ng/mL). (*P < 0.05; log-rank test). **(b)** The survival rate of *hif-1* mutant worms was not affected. (*P < 0.05; log-rank test). All experiments were repeated three biological times independently.

Since there is no successful animal model of oral lichen planus (OLP), we selected *C. elegans* as animal model for verification. We found that the lifespan of *C. elegans* treated with *P. gingivalis* LPS was shortened in a concentration-dependent manner. When the concentration reached 100 ng/mL, compared with the control group, the shortening of lifespan was statistically significant, but there was no significant difference between 1000 ng/mL and 100 ng/mL treatment groups. Therefore, we chose the concentration of 100 ng/mL for subsequent experiments (**Figure 6A**). To explore the role of *hif-1*, we also compared the effects of *P. gingivalis* LPS on wild-type *C. elegans* (N2) and *hif-1(ia4)* mutants under the same conditions. We found that 100 ng/mL *P. gingivalis* LPS decreased the lifespan in wild-type (N2) worms. However, 100 ng/mL *P. gingivalis* LPS did not further shorten the lifespan in *hif-1(ia4)* mutant worms (**Figure 6B**). These findings demonstrate that *P. gingivalis* LPS induces *C. elegans* death, in which the *hif-1* gene appears to play a key role in this process.

### *P. gingivalis* LPS causes accumulation of SQST-1 and LGG-1 proteins in *C. elegans*

**Figure 7:**
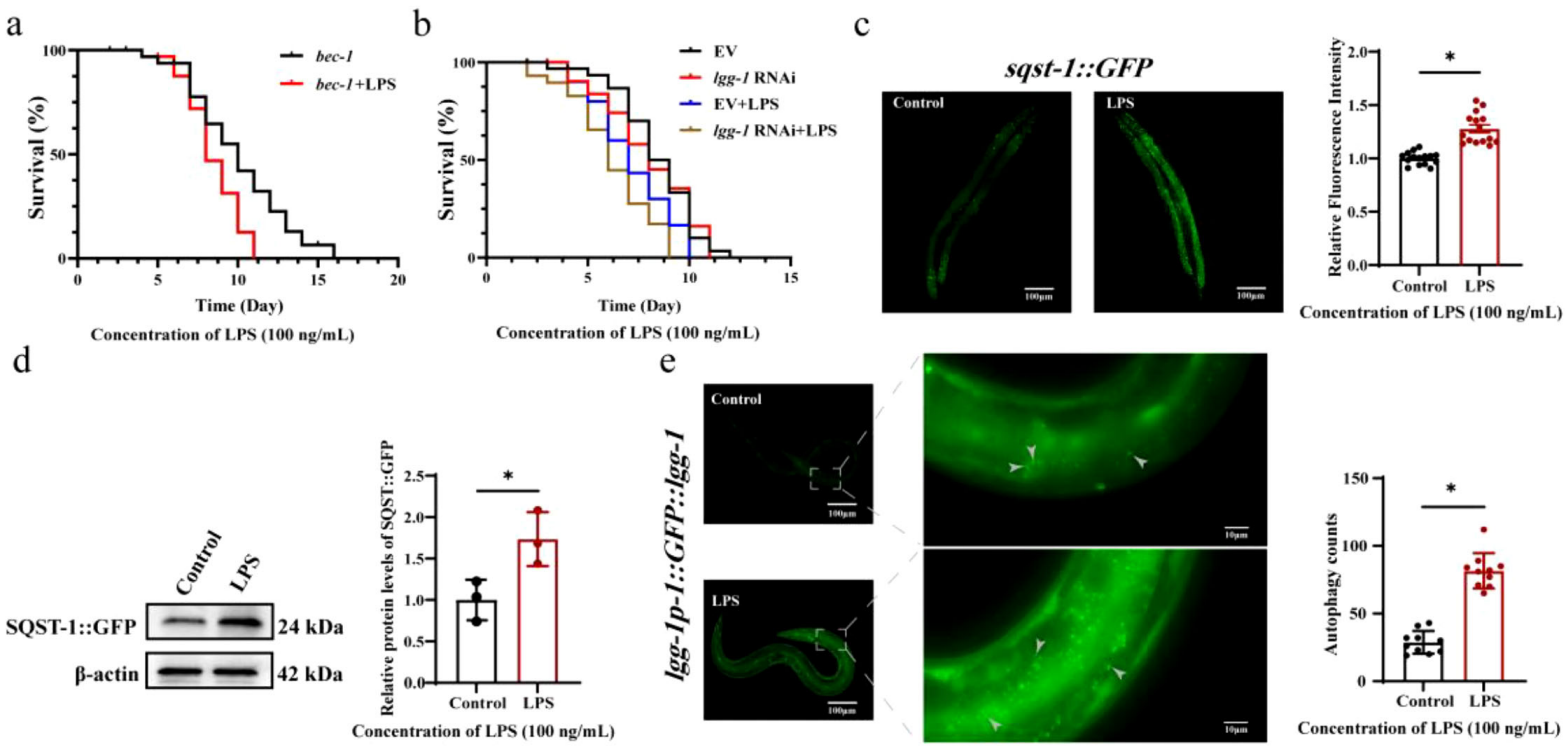
P*. gingivalis* LPS can induce significant accumulation of SQST-1 and LGG-1 proteins in *C. elegans*. **(a)** Survival of *bec-1* mutant worms exposed to *P. gingivalis* LPS. (*P < 0.05; log-rank test). **(b)** Survival of *lgg-1* RNAi worms exposed to *P. gingivalis* LPS. (*P < 0.05; log-rank test). **(c)** The levels of *sqst-1::GFP* after treatment with *P. gingivalis* LPS (100 ng/mL). Scale bars: 5 μm. N ≥ 15. (*P < 0.05, unpaired t-test). **(d)** The protein levels of SQST-1::GFP after treatment with *P. gingivalis* LPS (100 ng/mL). Data are presented as mean ± SD. The *P* value was calculated using the independent sample t test for the control group. **(d)** The levels of *lgg-1p-1::GFP::lgg-1* after treatment with *P. gingivalis* LPS (100 ng/mL). Scale bars: 0.5 μm. N = 10. (*P < 0.05, unpaired t-test). *P < 0.05. **P < 0.01. All experiments were repeated three biological times independently. **(e)** The effect of *P. gingivalis* LPS on the fluorescence expression of *lgg-1::GFP::lgg-1* autophagosome. The white arrow refers to the autophagosomes in *C.elegans*.

To study whether *P. gingivalis* LPS induces autophagy arrest in *C. elegans*, we conducted follow-up experiments. Survival assay data revealed that *P. gingivalis* LPS treatment shortened the lifespan of the *bec-1(ok691)* compared to the control group (**Figure 7A**). Likewise, the *lgg-1* RNAi exhibited reduced lifespan upon *P. gingivalis* LPS exposure (**Figure 7B**). It suggested that the loss of *bec-1* and *lgg-1* genes resulted in a significant increase in the sensitivity of *C. elegans* to *P. gingivalis* LPS, and they played an important role in host defense. Then, we observed *sqst-1::GFP* (homology p62) to assess changes in autophagy and found that *P. gingivalis* LPS increased the expression of *sqst-1::GFP* (**Figure 7C**). Western blot analysis additionally confirmed SQST-1 accumulation (**Figure 7D**). Moreover, the number of autophagosomes in *lgg-1p-1::GFP::lgg-1* (homology LC3) *C. elegans* was significantly higher in the *P. gingivalis* LPS-treated group than in the control group (**Figure 7E**). The present findings demonstrate that *P. gingivalis* LPS induces the accumulation of autophagy substrates and elevates autophagosome numbers in *C. elegans*, ultimately leading to autophagic functional impairment.

## Discussion

OLP is a chronic immune-inflammatory disease of unknown etiology, characterized by subepithelial lymphocytic infiltration and basal keratinocyte destruction (23). Despite ongoing research into this disease, its pathogenesis remains unclear. According to the existing research, it may be related to the cell-mediated immune process initiated by basal T lymphocytes (24). Previous studies have primarily focused on epithelial cell apoptosis induced by innate layer T cell-mediated immune responses, while little is known about the inflammatory and immune regulation of epithelial cells in OLP (25). In this study, by database analysis, we found that the lysosomal pathway exhibits abnormalities in OLP lesions, and the experimental results confirmed that most genes in this pathway show abnormal expression, leading to suppressed cellular activity. We also found that HIF-1α expression increased in the OLP inflammatory microenvironment and there was a certain correlation with the change of lysosomal in epithelial cells, suggesting that HIF-1α was closely related to lysosomal in OLP.

HIF-1α is a transcription factor that is difficult to detect under normal conditions, but it is highly expressed in hypoxia or inflammation of a variety of cells, and persistent inflammation can lead to increased oxygen consumption, resulting in local tissue hypoxia in the inflammatory site (26, 27). Hypoxia occurring within inflammatory lesions in turn promotes the stabilization of HIF-1α, thereby activating the transcription of target genes. In addition, it participates in the regulation of immune cell proliferation, apoptosis, differentiation, and autophagy (28, 29). Furthermore, HIF-1α modulates autophagy at various levels across different cell types (30). Research on OLP has revealed that compared to normal oral mucosal tissue, HIF-1α expression is significantly increased in OLP tissue (20). Moreover, HIF-1α polymorphisms enhance the activity of its target genes, thereby altering the OLP microenvironment and leading to the sustained release of inflammatory mediators (31). These findings imply that HIF-1α and its target genes may play a significant role in the pathogenesis of OLP. To clarify the molecular mechanism of HIF-1α in this study, we transfected si-HIF1A into HOK cells. After HIF-1α knockdown, the differences in gene expression along the lysosomal pathway and cellular activity gradually diminished compared to the control group. This confirms that HIF-1α regulates lysosomal in OLP, it is a previously unrecognized new function. However, it is not clear whether it can affect the autophagy of OLP epithelial cells by regulating lysosomal.

**Figure 8:**
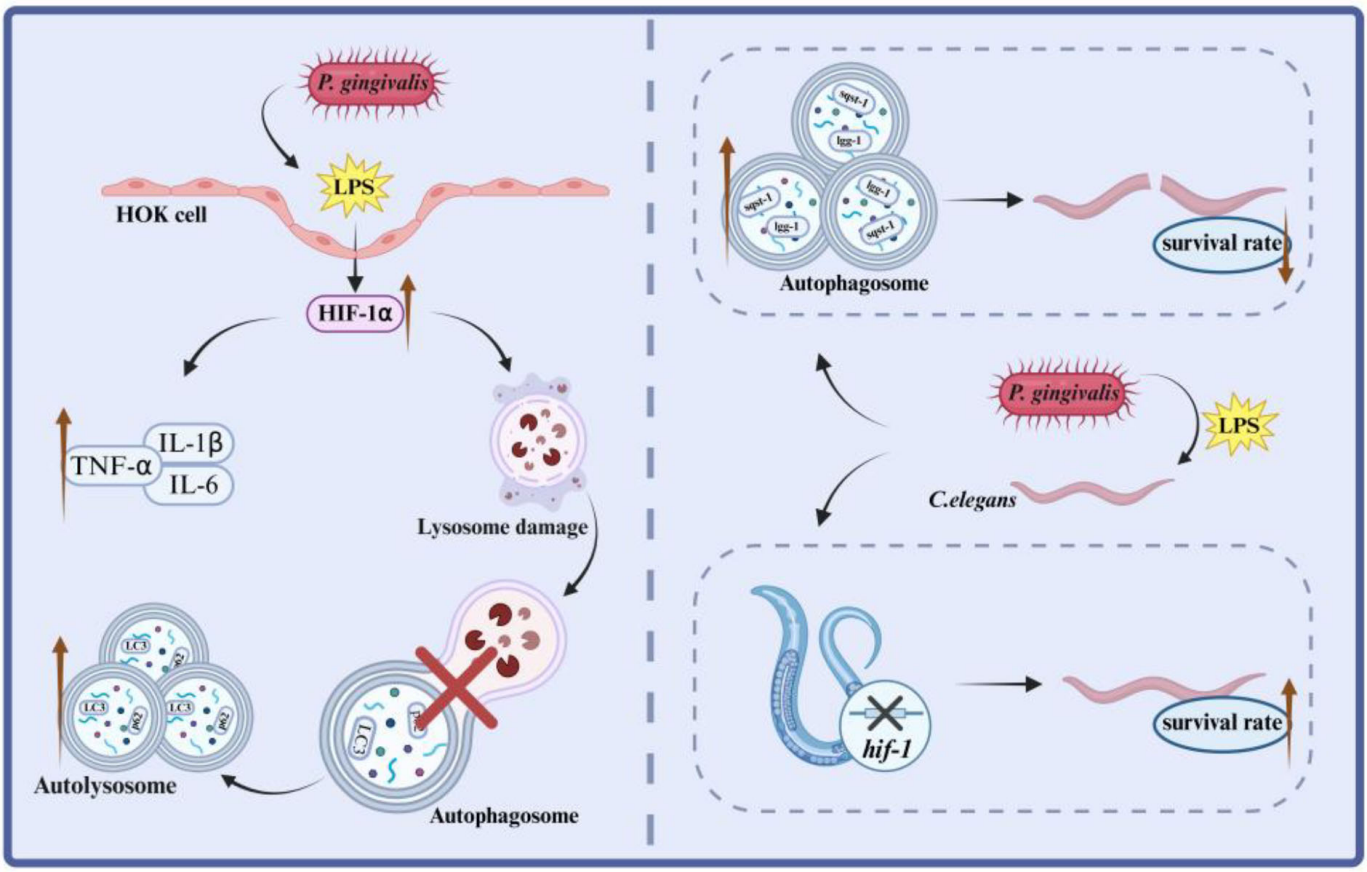
Schematic illustration of HIF-1α regulation of autophagy in oral keratinocytes and *C. elegans*.

Autophagy is a conserved lysosomal degradation pathway involved in cell development (32), antigen presentation, pathogen clearance and inflammation, it plays a crucial role in regulating innate immunity and adaptive immunity (33). On the one hand, autophagy not only participates in the clearance of pathogens and cell debris after apoptosis, but also plays a protective role against toxins, regulates the production of cytokines, and activates inflammasomes. On the other hand, autophagy is regulated by immune and stress signals. The coordinated interaction among these signaling pathways helps cells maintain homeostasis and physiological functions. Consequently, dysregulation of this network contributes to the pathogenesis of numerous diseases (34, 35). Current research indicates that T cells in the peripheral blood of OLP patients exhibit abnormal autophagy, which may be associated with immunopathological processes (34). Additionally, it was found that autophagy related 9B (ATG9B), as a key transmembrane protein for autophagosome formation, was up-regulated in oral lichen planus lesions and local T cells, especially in non-erosive oral lichen planus (36). These findings point to autophagy abnormalities occurring during the progression of OLP lesions. Interestingly, our results also showed that under the stimulation of *P. gingivalis* LPS, the autophagic substrate p62/SQSTM1 and the autophagosome marker protein LC3 in OLP showed significant accumulation, suggesting that the autophagic degradation link was blocked. In the *C. elegans* model, it was further confirmed that the deletion of autophagy core genes (*bec-1*, *lgg-1*) would aggravate the toxic effects induced by *P. gingivalis* LPS and shorten the lifespan of *C. elegans*. The expression of HIF-1α was significantly up-regulated under *P. gingivalis* LPS stimulation, and knockdown of HIF-1α could effectively restore the expression of autophagy-related genes and reduce the accumulation of autophagy marker proteins, which proved that HIF-1α was the key point to restore autophagy (**Figure 8**).

Although preliminary conclusions have been made in this study, the absence of established animal models analogous to OLP prevents us from providing additional *in vivo* experimental data. While *P.gingivalis* LPS stimulated-HOK provides a valuable and controllable system for dissecting molecular mechanisms, this model may not fully recapitulate the complex cellular interactions present in OLP lesions. Future studies using clinical samples are needed to validate our findings. Furthermore, how the HIF-1α specifically interferes with autophagy in OLP remains to be further explored. In summary, this study suggests that abnormal autophagy in OLP may form a positive feedback loop with the local inflammatory microenvironment, inflammatory signals inhibit autophagy through HIF-1α, leading to the accumulation of damaged proteins and organelles, This, in turn, exacerbates cellular stress and the release of inflammatory mediators, thereby contributing to pathological processes in OLP such as basal keratinocyte damage and subepithelial lymphocyte infiltration. Briefly, the autophagy disorder caused by HIF-1α plays a crucial role in the occurrence and development of OLP, potentially providing new insights into the pathogenesis of OLP.

## Materials and methods

### Cell culture

Normal human oral keratinocyte (HOK) derived from Otwo Biotech (China) was cultured with oral keratinocyte medium (ScienCell, USA) at 37 °C and 5% CO_2._ To simulate the inflammatory microenvironment of OLP *in vitro*, *P.gingivalis* lipopolysaccharide (Invivogen, France) was used to stimulate HOK. In the CCK-8 assay, cells were seeded in 96-well plates at a density of 5×10^3^ cells/well, and OD values were measured after *P.gingivalis* LPS stimulation for 24h. In the detection of inflammatory factors, the cells were seeded in a 6-well plate at a density of 3×10^5^ cells/well. After 24h of *P.gingivalis* LPS stimulation, RNA was extracted to detect the transcription level of inflammatory factors. After 45h, Brefeldin A 2.5 μg/mL was given to each well. Then after 4h, protein was extracted to detect the protein level of inflammatory factors. In separate experiments, si-HIF1A (Sangon, China) was transfected using Lipofectamine 2000 (Invitrogen, USA) to silence HIF-1α, HIF-1α siRNA target sequence: 5′-UUCCUCACACGCAAAUAGCdTdT-3′ (**Supplementary Table 3**). In this experiment, the cells were seeded on a 6-well plate at a density of 5×10^4^-1×10^5^ cells/mL, and transfected when the cell fusion degree reached 65%-70%. The concentration of HIF1A-siRNA solution was 20 nM. Next, the transcription level was detected at 24h after transfection, and the protein level was detected at 48h after transfection to verify the knockdown efficiency. The HOKs used in this experiment were the 3rd-6th generation cells after cell stabilization.

### Data collection and analysis

Download the dataset GSE52130 from Gene Expression Omnibus (GEO, https://www.ncbi.nlm.nih.gov/geo/). The R language limma package was used to compare the differences in gene expression levels between OLP samples and Control samples in the GEO dataset GSE52130. The screening conditions were: |log2FC| > 0.5, *P* < 0.05. A total of 2427 differentially expressed genes (DEGs) were screened, including 1182 up-regulated genes and 1245 down-regulated genes. To understand the molecular function and mechanism of differential genes in OLP, we performed Gene ontology (GO) and Kyoto Encyclopedia of Genes and Genomes (KEGG) enrichment analysis on the above differential genes. The screening conditions of GO enrichment analysis were FDR < 0.05, and the screening conditions of KEGG enrichment analysis were *P* < 0.05, then visual processing was performed. Because it is a public data set, information about the individual ‘s age and health status and drug use are not available, which seems to be a potential limitation.

### Transcription factor prediction

ChIP Atlas (https://chip-atlas.org/) and GTRD (http://gtrd.biouml.org/) are databases used for predicting transcription factors. We predicted potential transcription factors for DEGs in the lysosomal pathway online, following the detailed steps provided by each website. This gene set comprises 30 genes associated with lysosomal. Overlapping factors between the two databases were identified through Venn diagram analysis, with the Venn diagram generated using the online tool Venny 2. A total of 50 potential transcription factors were predicted (**Supplementary Table 1**).

### Reverse transcription-quantitative PCR

The total RNA of HOKs and *C. elegans* was extracted with TRIzol reagent (Invitrogen, USA), and the *C. elegans* samples were ground before adding TRIzol reagent. According to the manufacturer ‘s instructions, the first strand cDNA template was synthesized using the PrimeScript RT kit (TaKaRa). And SYBR Premix Ex kit (TaKaRa) was used for quantitative PCR. The relative number of transcripts was calculated according to the 2^-ΔΔCt^ formula. Gene expression was expressed relative to the reference gene *pmp-3 (*37–39). The PCR primer sequence is shown in **Supplementary Table 2**.

### Western blot

HOKs and *C. elegans* samples were lysed in RIPA lysis buffer containing 1 mM protease inhibitor on ice for 30 min and then centrifuged at 12000 rpm for 15 min at 4 °C. The supernatant was collected and quantitatively detected by BCA protein (Solarbio, China). The protein (15~30 μg) was loaded on 7.5% ~ 15% Bis-Tris SDS-polyacrylamide gel, 20 V electrophoresis for 10 min, 50 V electrophoresis for 30 min, and 90 V electrophoresis for 1 h. Then, the protein separated from the gel was transferred to a 0.22 μm PVDF membrane (Merck Millipore, Germany), 300 mA, for 90 min. The membrane was blocked with TBST (Tris-buffer salt solution 0.1% Tween 20) blocking solution containing 5% milk powder for 120 min, and the membrane was combined with IL-1β (1∶1000; CST), IL-6 (1:1000; CST), TNF-α (1:1000; Abcam), HIF-1α (1∶1000; Abcam), LC3 (1:1000; Abcam), SQSTM1/p62 (1:1000; Abcam) was incubated overnight at 4 °C, and β-actin antibody was used as the internal control (1:5000; Proteintech). The membrane was washed with TBST and then mixed with secondary antibody (1:5000; Proteintech) were incubated at room temperature for 1 hour. Protein bands were visualized using ECL reagents (Life-ilab, China), and quantitative analysis of protein band optical density was performed using ImageJ software.

### Cell viability assay

Cell viability was analyzed using CCK-8 (APExBIO, USA). 5×10^3^ cells were seeded in 96-well plates and treated with the specified reagent at the specified time point. Each well was added with 10 μL CCK-8 solution and incubated at 37 °C for 2 h to 3 h. The absorbance at 450 nm was measured by a microplate reader.

### Worm strains and cultivation

*C. elegans* maintains and reproduces under standard conditions (40–42), N2 Bristol wild-type, VC517 *bec-1(ok691)*, ZG31 *hif-1(ia4)*, MAH349 *sqst-1::GFP*, MAH215 *lgg-1p-1::GFP::lgg-1* were obtained from the Caenorhabditis Gpenetics Center (CGC).

### RNA interference

The strains of *E.coli* used for RNAi were obtained from the Ahringer library (43). *Unc-22* RNAi was included as a positive control in all experiments to account for RNAi efficiency. RNAi feeding experiments were performed on synchronized L1 to L2 larvae at 20 °C. In short, *E. coli* strains expressing dsRNA were grown overnight at 37 °C in LB broth containing 100 μg/ml ampicillin and then coated on NGM plates containing 100 μg/ml ampicillin and 5 mM isopropyl-1-thio-β-D-galactopyranoside (IPTG). The RNAi expression strain was cultured overnight at 25 °C (44, 45). The synchronized L1 to L2 larvae were placed on RNAi plates and cultured at 20 °C until maturity.

### Survival experiment

*E. coli* OP50 was cultured overnight at 37 °C in LB medium and coated on NGM plates. The synchronized *C. elegans* were cultured on *E. coli* OP50 at 20 °C until the young adult stage (i.e., within 12 h beyond the L4 stage). Approximately 50-60 worms were transferred onto NGM agar plates with or without 100 ng/mL *P. gingivalis* LPS and cultured at 25 °C. The number of living worms was counted at 24 h intervals. Immobile adult worms unresponsive to touch were scored as dead (46–48). Three plates were tested in each experiment, and three biological replicates were performed independently in all experiments.

### Fluorescence microscopy

The synchronized *sqst-1::GFP* and *lgg-1p-1::GFP::lgg-1* L1 worms were transferred to NGM plates with or without 100 ng/mL *P. gingivalis* LPS (49–51). After 24 hours, images were obtained using a Zeiss fluorescence microscope with a digital camera (Carl Zeiss, Jena, Germany). Fluorescence intensity and fluorescence quantity were quantified using ImageJ software. Each experiment tested 3 plates, about 40 nematodes per plate, and all experiments were performed independently for 3 times.

### Statistical analysis

Difference in survival rates was analyzed by the log-rank test. The results were considered significant with mean ± SD and *P* < 0.05. Independent sample t test and one-way ANOVA analysis of variance were performed using SPSS 29 (IBM, Armonk, New York).

## Data Availability

The original contributions presented in the study are included in the article/**Supplementary Material**. Further inquiries can be directed to the corresponding authors.
