# Supplementary material for: Hypoxia inducible factor 1 subunit alpha mediates autophagy disorder of oral lichen planus by regulating lysosomal pathway
Source: Front Immunol. 2026 Apr 14;17:1789261. doi: 10.3389/fimmu.2026.1789261 (PMC13121079; doi:10.3389/fimmu.2026.1789261)
Supplement: Supplementary file 1 [file DataSheet1.pdf]

Table S1 ChIP Atlas and GTRD Overlapping Transcription Factors

Gene name				
SMAD3	*SETDB1*	*AHR*	*TP53*	*AR*
RAD21	*BRD2*	*HIF1A*	*SPI1*	*CTCF*
ZEB1	*SMARCA4*	*RUNX1*	*MYC*	*SNAI2*
BRD4	*BMI1*	*HDAC1*	*FOS*	*JUNB*
HDAC2	*KLF4*	*SMC1A*	*POU5F1*	*KLF5*
RBPJ	*JUN*	*CREB1*	*NR2F2*	*IKZF1*
TFAP2C	*MITF*	*JUND*	*ATF3*	*SMC3*
FOXA2	*SMAD2*	*MAX*	*CDK9*	*EGR1*
SUZ12	*SMAD1*	*KMT2A*	*TP63*	*KDM1A*
EP300	*TEAD4*	*RNF2*	*ARNT*	*TBP*
