# Supplementary material for: Hypoxia inducible factor 1 subunit alpha mediates autophagy disorder of oral lichen planus by regulating lysosomal pathway
Source: Front Immunol. 2026 Apr 14;17:1789261. doi: 10.3389/fimmu.2026.1789261 (PMC13121079; doi:10.3389/fimmu.2026.1789261)
Supplement: Supplementary file 2 [file DataSheet2.pdf]

Table S2 Primer sequences involved in this work

Primer name	Forward (5′-3′)	Reverse (5′-3′)
hGAPDH	GGAGCGAGATCCCTCCAAAAT	GGCTGTTGTCATACTTCTCATGG
hLGMN	AACCCCAGAGAGTCGTCCTAC	ATCCAGTTGACGCTGTACCAG
hNEU1	CTTTGCTGAGGCGAGGAAAAT	TTGACAATGAACGCTGTAGGAG
hCTSK	ACTCAAAGTACCCCTGTCTCAT	CCACAGAGCTAAAAGCCCAAC
hCLTC	TGATCGCCATTCTAGCCTTGC	CTCCCACCACACGATTTTGCT
hGBA	CATCCGCACCTACACCTATGC	TGAGCTTGGTATCTTCCTCTGG
hIL-1β	TTCGACACATGGGATAACGAGG	TTTTTGCTGTGAGTCCCGGAG
hIL-6	ACTCACCTCTTCAGAACGAATTG	CCATCTTTGGAAGGTTCAGGTTG
hTNF-α	CCTCTCTCTAATCAGCCCTCTG	GAGGACCTGGGAGTAGATGAG
hMAP1LC3B	AAGGCGCTTACAGCTCAATG	CTGGGAGGCATAGACCATGT
hBECN1	ACCTCAGCCGAAGACTGAAG	AACAGCGTTTGTAGTTCTGACA
hSQSTM1	GCACCCCAATGTGATCTGC	CGCTACACAAGTCGTAGTCTGG
hATF3	CCTCTGCGCTGGAATCAGTC	TTCTTTCTCGTCGCCTCTTTTT
hCREB1	ATTCACAGGAGTCAGTGGATAGT	CACCGTTACAGTGGTGATGG
hNR2F2	TCATGGGTATCGAGAACATTTGC	TTCAACACAAACAGCTCGCTC
hSMAD3	TGGACGCAGGTTCTCCAAAC	CCGGCTCGCAGTAGGTAAC
hHIF1A	GAACGTCGAAAAGAAAAGTCTCG	CCTTATCAAGATGCGAACTCACA
pmp-3（*C.elegans*）	TGGATTGTCATTGGCGTCG	GTTGTCGCAGAGTGGTGTTT
lgg-1（*C.elegans*）	CCAGGACCATCACGAGGAAG	ACACATTCGTCGGCGGATAA
