# Supplementary material for: Hypoxia inducible factor 1 subunit alpha mediates autophagy disorder of oral lichen planus by regulating lysosomal pathway
Source: Front Immunol. 2026 Apr 14;17:1789261. doi: 10.3389/fimmu.2026.1789261 (PMC13121079; doi:10.3389/fimmu.2026.1789261)
Supplement: Supplementary file 3 [file DataSheet3.pdf]

Table S3 Si-HIF1A sequence

Primer name	Sequence (5'to3')
si-1	UCGACGUUCAGAACUUAUC/dT//dT/
si-2	UUCAGAUUCUUUACUUCGC/dT//dT/
si-3	UUCCUCACACGCAAAUAGC/dT//dT/
